# Estimation of Leaf Nitrogen Content in Wheat Based on Fusion of Spectral Features and Deep Features from Near Infrared Hyperspectral Imagery

**DOI:** 10.3390/s21020613

**Published:** 2021-01-17

**Authors:** Baohua Yang, Jifeng Ma, Xia Yao, Weixing Cao, Yan Zhu

**Affiliations:** 1 National Engineering and Technology Center for Information Agriculture/Collaborative Innovation Center for Modern Crop Production/Jiangsu Collaborative Innovation Center for the Technology and Application of Internet of Things, Nanjing Agricultural University, Nanjing 210095, China; ybh@ahau.edu.cn (B.Y.); majifeng@njau.edu.cn (J.M.); yaoxia@njau.edu.cn (X.Y.); caow@njau.edu.cn (W.C.); 2 School of Information and Computer, Anhui Agricultural University, Hefei 230036, China

**Keywords:** convolutional neural network, leaf nitrogen content, deep features, wheat, spectral features

## Abstract

Nitrogen is an important indicator for monitoring wheat growth. The rapid development and wide application of non-destructive detection provide many approaches for estimating leaf nitrogen content (LNC) in wheat. Previous studies have shown that better results have been obtained in the estimation of LNC in wheat based on spectral features. However, the lack of automatically extracted features leads to poor universality of the estimation model. Therefore, a feature fusion method for estimating LNC in wheat by combining spectral features with deep features (spatial features) was proposed. The deep features were automatically obtained with a convolutional neural network model based on the PyTorch framework. The spectral features were obtained using spectral information including position features (PFs) and vegetation indices (VIs). Different models based on feature combination for evaluating LNC in wheat were constructed: partial least squares regression (PLS), gradient boosting decision tree (GBDT), and support vector regression (SVR). The results indicate that the model based on the fusion feature from near-ground hyperspectral imagery has good estimation effect. In particular, the estimation accuracy of the GBDT model is the best (R^2^ = 0.975 for calibration set, R^2^ = 0.861 for validation set). These findings demonstrate that the approach proposed in this study improved the estimation performance of LNC in wheat, which could provide technical support in wheat growth monitoring.

## 1. Introduction

Wheat occupies an important position in agricultural production and strategic food reserves. Nitrogen is one of the main nutrients that affect wheat growth, yield and quality [1]. Therefore, rapid and accurate detection of the wheat nitrogen nutrition status is of great significance for guiding farmland management and improving wheat production efficiency, yield and quality [2].

At present, the implementation of remote sensing technology in precision agriculture provides new opportunities for the non-destructive real-time diagnosis of wheat nitrogen status and precise nitrogen control [3]. Among them, feature extraction has gradually become a key technology for non-destructive monitoring and diagnosis of crop nutrition, which has greatly expanded the feature expression ability of the crop canopy [4,5,6,7,8]. Previous studies have utilized many feature extraction methods, including principal component analysis (PCA) [9,10], neighborhood preserving embedding (NPE) [11], and linear discriminant analysis (LDA) [12]. In addition, full bands or characteristic bands extracted from the hyperspectral image have also been used to monitor crop growth, which obtained good estimation results. Leemans et al. successfully used the spectral features of the hyperspectral image to estimate the nitrogen content of wheat [13]. Mutanga et al. used the spectral features extracted by the depth analysis of the band to estimate the physiological and biochemical parameters of a variety of crops [14]. Although various methods of extracting spectral features have been proposed, the estimation model based only on spectral features has certain limitations. In particular, the inversion model of crop biochemical parameters based on spectral information is prone to saturation when the vegetation coverage is large [1]. Therefore, improving the accuracy of the estimation model still faces many difficulties.

Hyperspectral images (HSI) could provide not only spectral information, but also spatial information. Previous studies have shown that spatial feature extraction methods, such as discrete wavelet transform (DWT) [15,16], Gabor filtering [17,18], and local binary patterns (LBP) [19] have been successfully applied. However, most of the methods mentioned above closely rely on expert knowledge, which limits the predictive potential of the model [20]. Zheng et al. combined spectral features and spatial features to improve the effect of monitoring nitrogen nutrition in wheat [21]. However, facing the rapid growth of the information in hyperspectral images, it is difficult to achieve an optimal balance between typicality and robustness based on methods of feature extraction with prior knowledge. Therefore, it was still a big challenge to extract spatial features from HIS to monitor the nitrogen nutrition of wheat. 

In recent years, as an important branch of artificial intelligence, deep learning could solve complex problems with deep neural network models [22]. Moreover, the deep features extracted by deep learning methods have greatly improved the cognitive ability of the network [23,24]. Although the deep features are very abstract and most of them cannot be easily displayed, they are still being valued by more and more scholars. Pan et al. proposed a multi-grained scanning strategy to construct a multi-grained network (MugNet), which realized the extraction of deep features of hyperspectral images to improve the classification accuracy [25]. Chen et al. proposed a deep learning framework based on the fusion of spectral information and spatial information to extract the deep features of hyperspectral images [26]. Xu extracted the deep features of the spectrum to improve the detection of different types of Pu’er tea [27]. Yang et al. designed different structures of stacked autoencoder (SAE) to extract the deep features of hyperspectral images, which improved the accuracy of the estimation of soluble solid content in peach [28]. The research mentioned above showed that deep features could effectively improve the classification, recognition, and prediction of target objects.

In addition, as a representative algorithm of deep learning, CNN has received more and more attention due to its good results in the field of image detection. In comparison with traditional features, deep features are automatically learned layer by layer from spatial features through CNN, which is widely used in crop qualitative analysis. In particular, deep features were obtained based on CNN to detect wheat spikes [29] and crop nitrogen deficiency [30], as well as to obtain crop high-throughput phenotypic features [31,32]. The above research results show that CNN overcomes the limitations of traditional machine learning methods, which provides a new idea for HSI to estimate the leaf nitrogen content (LNC). As far as we know, the quantitative analysis of physiological and biochemical parameters in wheat using deep features extracted by CNN has not been reported in the literature.

Therefore, we used CNN to extract deep features from hyperspectral images of the wheat canopy, and constructed PLSR, SVR and GBDT models using deep features, spectral features, and fusion features to verify the estimation effects of different features. The purpose of this research was to (1) extract deep features from hyperspectral images to overcome the limitations of feature expression; (2) fuse spectral features and deep features to improve the estimation accuracy of the model; (3) evaluate the performance of different models to test the validity of the model

## 2. Data and Methods

### 2.1. Study Site and Experimental Design

**Figure 1:**
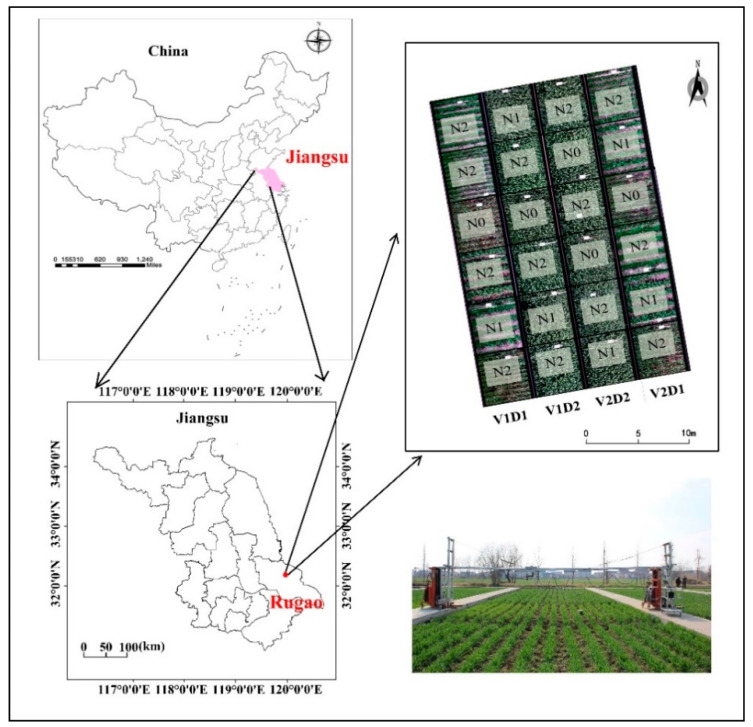
Location of the study area and hyperspectral imaging system. Note: D1 = 300 plants·m^−2^, D2 = 450 plants·m^−2^, N0 = 0 kg·N·ha^−1^, N1 = 150 kg·N·ha^−1^, N2 = 300 kg·N·ha^−1^, V1 = ‘Yangmai 18’, V2 = ‘Shengxuan 6’.

The experiments were carried out in Rugao Experimental Demonstration Base of the National Information Agriculture Engineering Technology Center in 2013 and 2014 (120°20′ E, 32°14′ N, Rugao City, Jiangsu Province), as shown in Figure 1. Rugao belongs to the subtropical monsoon climate zone. The annual average temperature and annual rainfall are 15.11 °C and above 1000 mm, respectively, which is very beneficial to the growth of wheat. The experiments were implemented in a total of 24 plots (7 m × 5 m for each plot), and the implementation of nitrogen fertilizer was based on three levels, including 0 (N0), 150 (N1) and 300 (N2) kg/ha, with two planting densities (300 plants·m^−2^ and 450 plants·m^−2^), as shown in Figure 1. The experimental varieties were ‘Yangmai 18’ and ‘Shengxuan 6’. Nitrogen fertilizer was applied according to 40% base fertilizer, 10% tiller fertilizer, 20% flower promoting fertilizer, and 30% flower retaining fertilizer. The basal fertilizer was combined with 135 kg/ha P_2_O_5_ phosphate fertilizer and 190 kg/ha K_2_O potassium fertilizer. The near-ground hyperspectral image and wheat plant samples were acquired simultaneously. The key periods of acquisition include the jointing, heading, flowering, and filling period.

### 2.2. Data Collection

All near-infrared hyperspectral images were collected with a pushbroom scanning sensor (V10E-PS, SpecIm, Oulu, Finland) mounted on a motorized rail. The sensor was about 1.0 m above the wheat canopy. The data obtained through the hyperspectral imaging system include hyperspectral images with 1392 × 1040 pixels and a total of 520 bands which range from 360 to 1025 nm with a spectral resolution of 2.8 nm. The spatial resolution and field of view for near-nadir observation were 1.3 mm and 42.8°. The original images were processed by the software specVIEW (SpecIm, Oulu, Finland) [33]. 

On the same day when the wheat canopy hyperspectral image was obtained in the field experiment, 20 wheat plants were randomly selected from each sampling area of the experimental base and brought back to the laboratory. First, the wheat was separated according to different organs (leaf, stem, and ear) as experimental samples. Secondly, all samples were placed in an oven at 105 °C for 30 min and dried at 80 °C for more than 20 h. Finally, they were weighed to obtain the dry weight of each sample. The samples were crushed and the N content in the leaf, stem and ear was separately determined by the Kjeldahl method [34].

### 2.3. Spectral Features and Deep Features

#### 2.3.1. Vegetation Indices

Vegetation indices are the combination of linear and non-linear features in the visible–near-infrared band, which is one of the most widely used indicators in crop growth monitoring, and reflects the growth of crops under certain conditions. In this study, 26 VIs related to LNC in wheat were selected, for which calculation formulas are shown in Table 1.

Where R is the reflectance. I, II, III, IV, and V are only used to distinguish the same vegetation indices of different wave bands.

#### 2.3.2. Position Features

The crop canopy has strong absorption and reflection characteristics in the visible and near-infrared bands, which are related to the physiological and biochemical components of the crop [60]. To enhance the absorption characteristics of LNC and remove the influence of soil and other background spectrum absorption, the continuum removal method was used to process the canopy reflectance spectrum in wheat.

**Figure 2:**
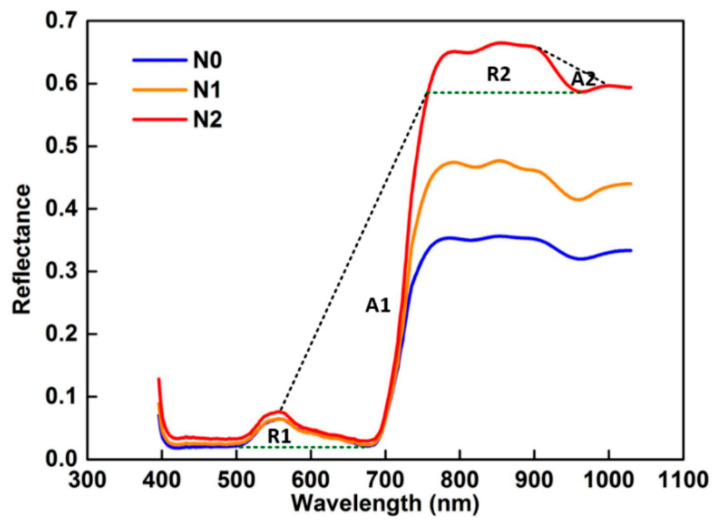
Characteristic absorption and reflection positions of the winter wheat for the three nitrogen treatments. (A) Absorption position, and (R) reflection position.

In this study, the ENVI software was used to extract the spectral information (400–1000 nm) of the hyperspectral image, and then the continuum removal method was used to further extract the spectral position features, which generally include absorption characteristic and reflection characteristic [61]. The spectral reflectance curve and the continuum removal curve are shown in Figure 2. It can be seen from Figure 2 that the reflection characteristic bands are distributed at 500–721 and 753–959 nm, and the spectral absorption characteristic bands are distributed at 557–754 and 900–1030 nm. Within the green dotted line and the black dotted line are the reflection and absorption positions of the spectral features, respectively. The calculation formulas of the six characteristic parameters are shown in Table 2 [61,62].

$R_{\mathrm{ci}}$ is the continuum removal curve, $R_{i}$ is the original reflectance curve, λ is the wavelength, $\lambda_{j}$ and $\lambda_{k}$ are the initial and final wavelengths of each absorption and reflection region, respectively, and the index i is the number of the corresponding band.

In addition, position features also include band position features, which include the red edge position, yellow edge position, and blue edge position, etc. The waveband position parameters used in this study are shown in Table 3, with a total of 13 features [61]. 

#### 2.3.3. Deep Features

In this study, deep features were extracted using a convolutional neural network (CNN), which is a deep feedforward artificial neural network including a convolutional layer, pooling layer, and fully connected layer [30]. Due to the large number of features extracted by CNN and the relatively small dataset used in this study, the model is prone to overfitting if the data are directly input to the network for training. Therefore, a transfer learning method (a machine learning method) was used to extract deep features from hyperspectral images using the trained CNN named AlexNet, which is a convolutional neural network proposed by Krizhevsky et al. in 2012, who designed and applied the network, and won the championship in the ILSVRC competition [63]. Moreover, AlexNet was trained on a subset of the large ImageNet database.

**Figure 3:**
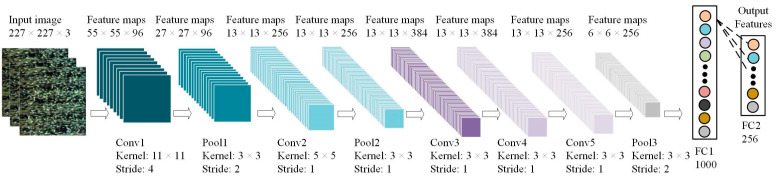
Convolutional neural network structure. Kernel represents the size of the convolution kernel (pixels), Stride represents the sliding step size, Conv1, Conv2, Conv3, Conv4, Conv5 represent the first, second, third, fourth, and fifth convolutional layers, and Pool1, Pool2, and Pool3 represent the first, second, and third pooling layer, FC1 and FC2 represent fully connected layer.

The convolutional neural network model is shown in Figure 3. It can be seen from Figure 3 that the number of feature maps and kernels in each layer is different, and the structure of CNN (AlexNet) includes five convolutional layers, three pooling layers and two fully connected layers. A total of 256 dimension feature vectors are extracted through FC2 as deep features. This experiment was performed based on a Pytorch framework with Windows system. The specific parameters are as follows: an Intel Core i7-8700 @ 3.20 GHz × 6, a memory of 16 GB, and a GPU of Nvidia GeForce RTX 2080.

### 2.4. Feature Optimization Method

#### 2.4.1. Random Forest Algorithm

To obtain features which have a higher contribution to the estimation model, random forest (RF) was used to optimize the features. The random forest algorithm is a multi-classifier algorithm based on ensemble learning, which consists of a decision tree and a bagging algorithm [64]. Bagging is a process in which sub-sampling (collected samples) is put back into random sampling again [65]. Then, each round of different random sampling is used for model training, and other unsampled data are used as out-of-bag (OOB) to verify the model. Generally, random sampling twice can ensure that the model has strong generalization [64]. RF-based feature selection is mainly achieved by directly measuring the influence of each feature on the accuracy of the model by reducing the average accuracy. 

#### 2.4.2. Pearson Correlation Coefficient Method

Pearson correlation analysis is used to obtain the correlation coefficient (*r*) between variables, which reflects the degree of correlation. For two vectors X and Y of the same dimension, the Pearson correlation coefficient calculation formula is as follows:

(1) $$r=\frac{\sum \left(X-\bar{X})(Y-\bar{Y}\right)}{\sqrt{\sum {\left(X-\bar{X}\right)}^{2}{\left(Y-\bar{Y}\right)}^{2}}}$$

where, $\bar{X}$ is the mean of X, and $\bar{Y}$ is the mean of Y.

The value of *r* ranges from −1 to 1. When *r* is less than zero, it means that the two vectors are linearly negatively correlated. However, in terms of evaluating the importance of features, the correlation between two vectors has been paid more attention, and its absolute value (|r|) shows the strength of the correlation. 

### 2.5. Regression Method

#### 2.5.1. Partial Least Squares Regression

Partial least squares (PLS) is a multivariate statistical data analysis method. Partial least squares regression (PLSR) is an extended form of multiple linear regression model, which combines multiple linear regression, canonical correlation analysis, and principal component analysis, and extracts principal components through variable mapping methods. PLSR can avoid multiple linear relationships between independent variables and dependent variables, which can improve the accuracy, robustness, and practicability of the model [66].

#### 2.5.2. Support Vector Regression

Support vector regression (SVR) is based on the principle of structural risk minimization, which uses a small number of support vectors to represent the entire sample set [67]. When the SVR model is established, different parameters need to be set including the penalty coefficient C and the kernel parameter g. It is easy to cause over-fitting and under-fitting of the model if the value of C is too large or too small, and g determines the distribution of the data after mapping to the new feature space. The value of *g* indicates the number of support vectors. 

#### 2.5.3. Gradient Boosting Decision Tree

Gradient boosting decision tree (GBDT) is an important algorithm in ensemble learning, which combines multiple decision trees to build a more powerful model [68]. GBDT can effectively perform regression estimation by constructing and combining multiple learners to complete the learning task, which is usually more sensitive to parameter settings. Among them, a more important parameter is learning_rate, which is used to control the strength of each tree to correct the error of the previous tree. A higher learning_rate means that each tree can make stronger corrections, which makes the model more complicated. 

In this study, the data obtained in 2013 were used as the calibration set of the model, and the data obtained in 2014 were used as the validation set. The coefficient of determination (R^2^) and root mean square error (RMSE) were used as evaluation indicators to evaluate all established regression models, which include SVR, GBDT and PLSR. Generally, universal models have relatively higher R^2^ and smaller RMSE [69].

## 3. Results and Analysis

### 3.1. Optimization of Vegetation Indices

**Figure 4:**
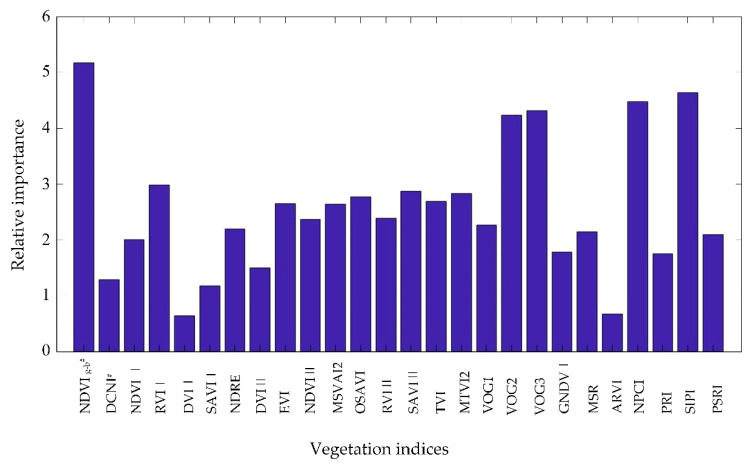
Feature importance of vegetation indices based on the random forest algorithm.

To reduce the information redundancy and improve the accuracy of the model, the random forest algorithm was used to analyze the relative importance of 26 vegetation indices, as shown in Figure 4. The relative importance of the vegetation indices was ranked in descending order, and the top 30% are selected as the preferred VIs, which are NDVI _g-b_^#^, SIPI, NPCI, VOG3, VOG2, RVI I, SAVI II and MTVI2.

### 3.2. Optimization of Position Features

**Figure 5:**
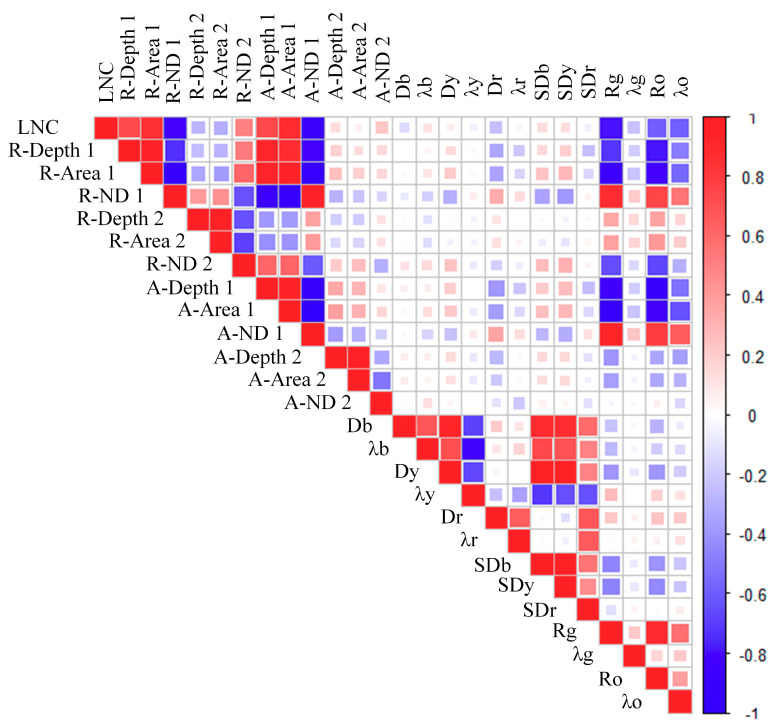
Matrix of correlation coefficient between leaf nitrogen content (LNC) and position features.

To extract sensitive position features, the correlation coefficients of 25 position features with LNC were calculated using Pearson correlation coefficient method. The results are shown in Figure 5. The first row in the triangular matrix represents the correlation between position features and LNC, and the rest represent the correlation between position features. The first 30% of the larger absolute value of the correlation coefficient are selected as the preferred position features, including Rg, R_Depth1, R_Aear1, R_ND1, A_Depth1, A_Aear1, A_ND1, and the corresponding correlation coefficients are −0.79, 0.73, 0.85, −0.81, 0.73, 0.87, and −0.89, respectively. 

### 3.3. Optimization of Deep Features

**Figure 6:**
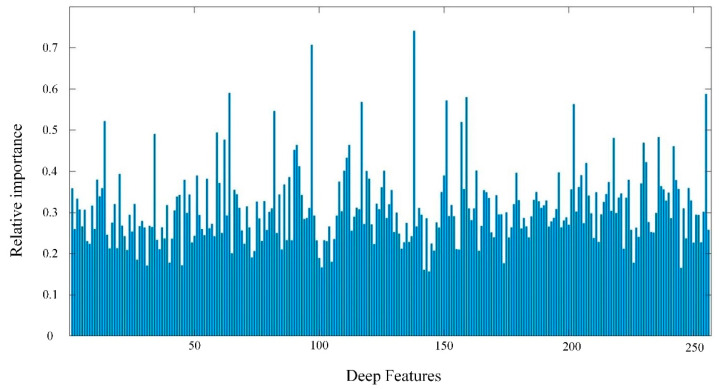
Optimal selection of deep features based on random forest algorithm.

The hyperspectral image of wheat canopy with 256 dimensional deep features is extracted by convolutional neural network. To more effectively estimate LNC of wheat, random forest algorithm was used to optimize deep features. As shown in Figure 6, the relative importance of these deep features is different. The first 20 features with relative importance greater than 0.45 were selected and merged with the spectral features for subsequent modeling research.

### 3.4. Comparison of Models for Estimating LNC in Winter

Different features were obtained from the near-ground imaging spectroscopy of wheat canopy based on different methods, including spectral features and deep features, to construct different models for estimation LNC, which includes PLS, GBDT, and SVR models. The results of the model comparison are shown in Table 4. For the PLS model, R^2^ ranges from 0.791 to 0.895 for the calibration set and from 0.708 to 0.814 for the validation set. For the SVR model, R^2^ is 0.791–0.954 and 0.659–0.842. For the GBDT model, R^2^ is 0.848–0.975 for the calibration set and 0.717–0.861 for the validation set. The results show that the estimations based on the PLS, GBDT and SVR models all perform well.

Where VIs represents vegetation indices, PFs represents position features, DFs represents deep features, DFs represents deep features, and FFs represents fusion features.

**Figure 7:**
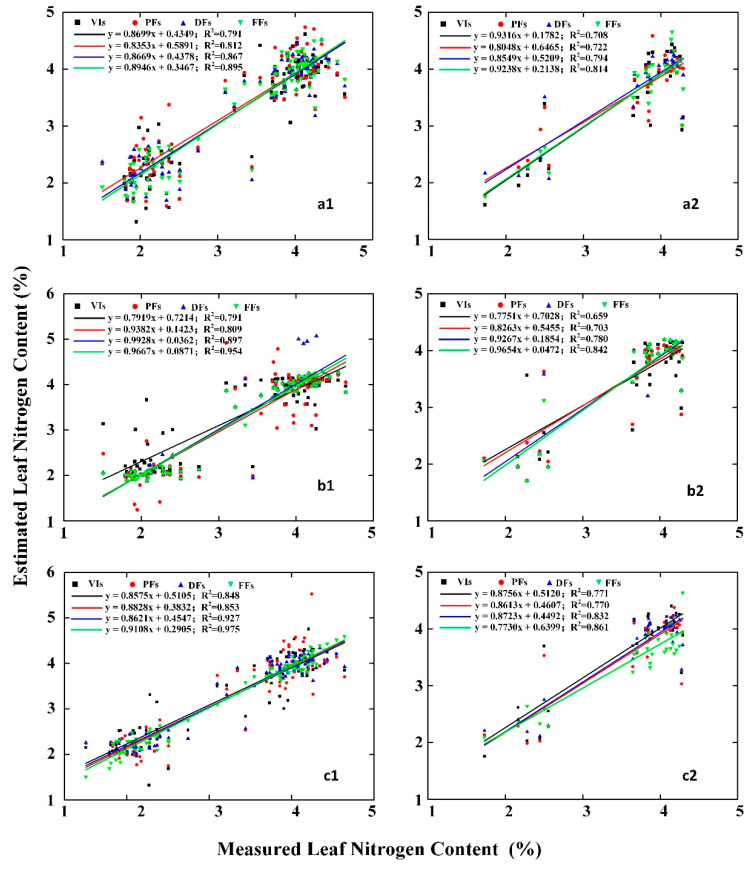
Estimated and measured leaf nitrogen content (%) in wheat. Left: validation set, right: calibration set with PLS (**a1**,**a2**), SVR (**b1**,**b2**), and GBDT (**c1**,**c2**). VIs: vegetation indices, PFs: position features, DFs: deep features, FFs: fusion features.

From the comparison of the models based on different features shown in Figure 7, we could find that the R^2^ of the model ranges from 0.791 to 0.848 based on VIs, from 0.809 to 0.853 based on PFs, from 0.867 to 0.927 based on DFs, and from 0.895 to 0.975 based on FFs for the calibration set. The corresponding R^2^ of the model are 0.659–0.717, 0.703–0.77, 0.78–0.832, and 0.814–0.861 for the validation set. Therefore, regardless of the validation set or calibration set, the estimation results of model with a combination of features perform better than a single feature.

## 4. Discussion

### 4.1. Deep Features and Spectral Features

With the improvement of the ability to collect and store data, this has brought about many difficulties in data reduction and analysis. Feature extraction methods for higher-resolution spectral information are becoming increasingly important. In this study, the vegetation index provided important information for quantifying wheat LNC. However, only a few wavelengths of reflectance are used in the vegetation index, which affects the robustness of the model for monitoring wheat nitrogen nutrition [70]. In order to highlight the difference between the spectral absorption features of LNC, the continuum removal transform is used to mine more potential information of spectral position features, which can not only solve the saturation problem of the existing index, but also effectively reduce the influence of the background on the spectral features [14].

Moreover, the expression of features in high-dimensional data is also increasingly important, especially for non-linear features. Traditional features only focus on fewer and relatively obvious features, and these features are not reliable for different input data. In contrast, the deep features extracted from hyperspectral images based on deep learning could express the detailed information of spatial features. To estimate wheat LNC more accurately, spectral information and spatial information were extracted to realize the comprehensive features of hyperspectral images, which overcome the limitations of the traditional single feature.

### 4.2. The Necessity of Extracting Deep Features from Hyperspectral Images

Previous studies have shown that convolutional neural networks (CNN) can extract deep features. The feature extractor at each layer can use the convolutional layer and the pooling layer to convert the input raw data to complex deep features, thereby reducing the data noise problem caused by external environmental interference [71]. Especially when traditional methods cannot collect enough features to support accurate detection, CNN can still extract more detailed features, which will help to increase the detection potential [23]. Cheng et al. proposed a deep learning model that combines spectral and spatial features, which can effectively extract complex hyperspectral features [72]. Therefore, to extract the features which play a key role in the quantitative analysis of the nitrogen content of wheat leaves from the hyperspectral image, it is necessary to maintain the spatial topological structure of the hyperspectral image of the wheat canopy, and the deep features are extracted layer by layer through CNN and gradually abstracted based on the constructed convolutional neural network. 

Although the CNN method can fully obtain the high-dimensional deep features of the sample, not all of the detected deep features are useful, and the high-dimensional data lead to the complexity of calculation and analysis [73]. In particular, the excessive increase in the network depth of the convolutional neural network will lead to some negative effects, such as overfitting, disappearance of gradients, and decreased accuracy [74]. Therefore, to solve the general problem of imbalance between the limitation and availability of high-dimensional deep features, the random forest algorithm was used to eliminate irrelevant and redundant features of deep features. Thus, the complexity of the model was reduced, and the accuracy and generalization ability of the model were improved. 

### 4.3. Different Models and Different Features

Fusion features including deep features and spectral features extracted from hyperspectral imagery can successfully estimate wheat LNC. Fan et al. used the position features and vegetation index to estimate wheat LNC [61]. However, the extracted features only included spectral features. In this study, not only the spectral features (VIs and PFs) were extracted, but also the spatial features (deep features) were extracted based on the convolutional neural network. Table 2 showed that the accuracy of the GBDT model based on fusion features was 8.2% higher than that of the PLS model, and 2.2% higher than that of the SVR model. 

In addition, the estimation performances of different regression models (PLSR, SVR, GBDT) were good. Specially, the effect based on the GBDT model was better than the other two regression models. Despite the experimental design of different models and different features, the proposed model achieved good estimation results. In the future, it is necessary to collect more data to verify the method proposed in this work, which can provide technical support for estimating wheat LNC.

## 5. Conclusions

In this study, a method based on fusion features was proposed to estimate wheat LNC, including spectral features and deep features. On the one hand, fusion features include both spectral features and spatial features, and the extraction of spatial features (deep features) was based on CNN, which could learn spatial hierarchy, including basic features and semantic features. On the other hand, PLS, GBDT, and SVR models were constructed to estimate wheat LNC. The GBDT model has the highest accuracy (R^2^ = 0.975 for calibration set, R^2^ = 0.861 for validation set), which provides a new approach for quantitative estimation of wheat LNC from hyperspectral imagery. In addition, it is necessary to further try more deep learning models in future research to provide technical support for crop growth monitoring.

## Figures and Tables

**Table 1:** The calculation formulas for vegetation indices.

Index	Formula	Reference
$NDVI_{g-b}{}^{\#}$	$\left(R_{573}-R_{440}\right)/\left(R_{573}+R_{440}\right)$	[35]
$DCNI^{\#}$	$\left(R_{720}-R_{700}\right)/\left(R_{700}-R_{670}\right)/\left(R_{720}-R_{670}+0.03\right)$	[36]
NDVI I	$\left(R_{800}-R_{670}\right)/\left(R_{800}+R_{670}\right)$	[37]
RVI I	$R_{800}/R_{670}$	[38]
DVI I	$R_{800}-R_{670}$	[39]
SAVI I	$1.5\times \left(R_{800}-R_{670}\right)/\left(R_{800}+R_{670}+0.5\right)$	[40]
NDRE	$\left(R_{790}-R_{720}\right)/\left(R_{790}+R_{720}\right)$	[41]
DVI II	$R_{NIR}-R_{R}$	[42]
EVI	$\frac{2.5\times \left(R_{NIR}-R_{R}\right)}{\left(R_{NIR}+6R_{R}-7.5R_{B}+1\right)}$	[43]
NDVI II	$\left(R_{NIR}-R_{R}\right)/\left(R_{NIR}+R_{R}\right)$	[44]
MSAVI2	$\left(2R_{NIR}+1-sqrt\left({\left(2R_{NIR}+1\right)}^{2}-8\left(R_{NIR}-R_{R}\right)\right)\right)/2$	[45]
OSAVI	$\frac{\left(1+0.16\right)\left(R_{NIR}-R_{R}\right)}{R_{NIR}+R_{R}+0.16}$	[46]
RVI II	$R_{NIR}/R_{R}$	[47]
SAVI II	$\frac{1.5\times \left(R_{NIR}-R_{R}\right)}{R_{NIR}+R_{R}+0.5}$	[48]
TVI	$60\times \left(R_{NIR}-R_{G}\right)-100\times \left(R_{R}-R_{G}\right)$	[49]
MTVI2	$\frac{1.5*\left(1.2*\left(R_{NIR}-R_{G}\right)-2.5*\left(R_{R}-R_{G}\right)\right)}{\sqrt{\left({\left(2*R_{NIR}+1\right)}^{2}-\left(6*R_{NIR}-5*\sqrt{\left(R_{R}\right)}-0.5\right)\right)}}$	[50]
GNDVI	$\left(R_{NIR}-R_{R}\right)/\left(R_{NIR}+R_{R}\right)$	[51]
MSR	$\left(R_{NIR}/R_{R}-1\right)/\left(R_{NIR}/R_{R}+1\right)$	[52]
ARVI	$\frac{R_{NIR}-R*B}{R_{NIR}+R-\left(B-R\right)}$	[53]
VOG1	$R_{740}/R_{720}$	[54]
VOG2	$\left(R_{734}-R_{747})/(R_{715}+R_{726}\right)$	[54]
VOG3	$\left(R_{734}-R_{747})/(R_{715}+R_{720}\right)$	[54]
PRI	$\left(R_{531}-R_{570}\right)/\left(R_{530}+R_{570}\right)$	[55,56]
NPCI	$\left(R_{680}-R_{430}\right)/\left(R_{680}+R_{430}\right)$	[57]
SIPI	$\left(R_{800}-R_{445}\right)/\left(R_{800}-R_{680}\right)$	[58]
PSRI	$\left(R_{680}-R_{500}\right)/R_{750}$	[59]

**Table 2:** Characteristic parameters for absorption and reflection positions.

Variables	Calculation Formula
$A\text{-}Depth_{i}$	$1-R_{i}(\lambda_{\min})/R_{\mathrm{ci}}(\lambda_{\min})$
$A\text{-}Area_{i}$	$\int_{\lambda_{j}}^{\lambda_{k}}(R_{\mathrm{ci}}(\lambda )-R_{i}(\lambda ))d\lambda$
$A\text{-}ND_{i}$	$A\text{-}Depth_{i}/A\text{-}Area_{i}$
$R\text{-}Depth_{i}$	$1-R_{\mathrm{ci}}(\lambda_{\max})/R_{i}(\lambda_{\max})$
$R\text{-}Area_{i}$	$\int_{\lambda_{j}}^{\lambda_{k}}(R_{i}(\lambda )-R_{\mathrm{ci}}(\lambda ))d\lambda$
$R\text{-}ND_{i}$	$R\text{-}Depth_{i}/R\text{-}Area_{i}$

**Table 3:** Definition and description of waveband position parameters.

Variables	Names	Definition and Description
Db	Blue edge amplitude	Maximum value of the 1st derivative of a blue edge (490–530 nm)
λb	Blue edge position	Wavelength at Db
Dy	Yellow edge amplitude	Maximum value of the 1st derivative of a yellow edge (560–640 nm)
λy	Yellow edge position	Wavelength at Dy
Dr	Red edge amplitude	Maximum value of the 1st derivative with a red edge (680–760 nm)
λr	Red edge position	Wavelength at Dr
Rg	Green peak amplitude	Maximum reflectance of a green peak (510–560 nm)
λg	Location of green peak	Wavelength at Rg
Ro	Red valley amplitude	Lowest reflectance of a red well (650–690 nm)
λo	Red valley position	Wavelength at Ro
SDb	Blue-edge integral areas	Sum of the 1st derivative values within the blue edge
SDy	Yellow-edge integral areas	Sum of the 1st derivative values within the yellow edge
SDr	Red-edge integral areas	Sum of the 1st derivative values within the red well

**Table 4:** Estimating model for wheat LNC from the selected input variables with three machine learning techniques.

Model	Features	Preferred	Calibration Set		Validation Set	
		Variables	R^2^	RMSE	R^2^	RMSE
PLS	VIs	8	0.791	0.448	0.708	0.439
	PFs	7	0.812	0.421	0.722	0.392
	DFs	20	0.867	0.352	0.794	0.330
	FFs	35	0.895	0.313	0.814	0.328
SVR	VIs	8	0.791	0.442	0.659	0.449
	PFs	7	0.809	0.448	0.703	0.416
	DFs	20	0.897	0.325	0.780	0.367
	FFs	35	0.954	0.209	0.842	0.312
GBDT	VIs	8	0.848	0.148	0.717	0.384
	PFs	7	0.853	0.137	0.77	0.386
	DFs	20	0.927	0.084	0.832	0.303
	FFs	35	0.975	0.01	0.861	0.263

## Data Availability

Restrictions apply to the availability of these data. Data was obtained from Jiangsu Key Laboratory for Information Agriculture and are available from the authors with the permission of Jiangsu Key Laboratory for Information Agriculture.
